# GBS Data Identify Pigmentation-Specific Genes of Potential Role in Skin-Photosensitization in Two Tunisian Sheep Breeds

**DOI:** 10.3390/ani10010005

**Published:** 2019-12-18

**Authors:** Imen Baazaoui, John McEwan, Rayna Anderson, Rudiger Brauning, Alan McCulloch, Tracey Van Stijn, Sonia Bedhiaf-Romdhani

**Affiliations:** 1 Faculty of Science of Bizerte, University of Carthage, Carthage 1054, Tunisia; 2 AgResearch Ltd., Invermay Agricultural Centre; Mosgiel 9092, New Zealand; 3 National Agricultural Research Institute of Tunisia, Laboratory of Animal and forage Production, University of Carthage, Ariana 1004, Tunisia

**Keywords:** genotyping-by-sequencing (GBS), pigmentation genes, Photosensitization, Tunisian sheep

## Abstract

**Simple Summary:**

Maintaining the flexibility of the genetic resources of native animals to face local environment constraints is still a major challenge. In Tunisia, the Noire de Thibar breed is a local sheep, typically with black coloration, known for its ability to tolerate “*hypericum perforatum*”, which causes skin photosensitization in white colored sheep. The goal of this study was to perform a genome scan, by considering genotyping-by-sequencing (GBS) markers that were genotyped in divergent coat colored sheep (black vs. white) to identify strong, and recent, artificial selection that is involved in skin-photosensitization. Interestingly, the genomic differentiation analysis identified F_ST_ markers within genomic regions containing key pigmentation and photosensitivity related-genes. These findings help in understanding the background of coat color genetics and its potential role in adaptation to local environment constraints.

**Abstract:**

The Tunisian Noire de Thibar sheep breed is a composite breed, recently selected to create animals that are uniformly black in order to avoid skin photosensitization after the ingestion of toxic “*hypericum perforatum*” weeds, which causes a major economic loss to sheep farmers. We assessed genetic differentiation and estimated marker F_ST_ using genotyping-by-sequencing (GBS) data in black (Noire de Thibar) and related white-coated (Queue fine de l’ouest) sheep breeds to identify signals of artificial selection. The results revealed the selection signatures within candidate genes related to coat color, which are assumed to be indirectly involved in the mechanism of photosensitization in sheep. The identified genes could provide important information for molecular breeding.

## 1. Introduction

Patterns of genetic variation processes in domestic animals have long proven insightful for the study of domestication, breed formation, population structure, and the consequences of selection [1]. Natural and artificial selection processes in domestic animals play crucial roles in maintaining the flexibility of animal genetic resources in facing local environment constraints. In livestock ruminants, skin photosensitization, caused by the ingestion of toxic plants, is relatively common and affects animal production. In the North of Tunisia, sheep farmers face major economic losses from the intoxication of white sheep following the ingestion of ‘*hypericum perforatum*’ or ”*St. John’s wort*” weeds, with animals frequently showing photosensitivity symptoms [2]. Indeed, this plant contains *hypercin* that causes a photosensitive anaphylactic reaction in animals with white-colored skin resulting in high mortality [3,4,5]. Therefore, a local sheep breed called ’Noire de Thibar’ or ‘Black Thibar’ has been specifically created to tolerate skin photosensitivity through a crossbreeding program between local white-coated Queue fine de l’ouest and black French Merinos d’Arles breeds to create animals that are uniformly black [6,7,8]. Furthermore, a new gene pool was introduced into the breed using the Black Brown Swiss breed in order to fix the black coat color, to avoid consanguinity, and to improve meat and wool quality [6,9,10]. In addition, the uniform black wool color is in high demand for traditional carpet and clothing manufacturers. Many studies have reported the role of melanocytes that specialize in producing melanin pigments in protecting organisms from ultraviolet radiation [11,12]. In humans, several genomic association studies have evidenced the effect of pigmentation-related-genes on photosensitivity symptoms and skin pigment disorders [13,14]. The advent of high throughput and cost-effective genotyping techniques allows for the evaluation of the response, at the genome level, to various selective pressures in a wide range of adaptive traits. For example, genotyping-by-sequencing (GBS) on livestock has been a sufficient solution that is required for population genetic and genomic studies [15]. This method has been successfully used to identify selection signatures of important economic traits in cattle [16], pigs [17,18], chicken [19], and camels [20]. The goal of this investigation was to perform a pair-wise marker differentiation comparison in a genome-wide scan between Noire de Thibar and Queue fine de l’ouest Tunisian sheep breeds, using genotyping-by-sequencing data to reveal putative regions under strong and recent selection.

## 2. Materials and Methods

### 2.1. Ethics Statement

The blood used for all of the analyses was collected by veterinarians during routine blood sampling ofcommercial farm animals (for medical care or follow up). These animals were not linked to any experimental trials. All the samples and data processed in our study were obtained with the breeders and breeding organizations’ consent.

### 2.2. Sheep Breeds

**Figure 1:**
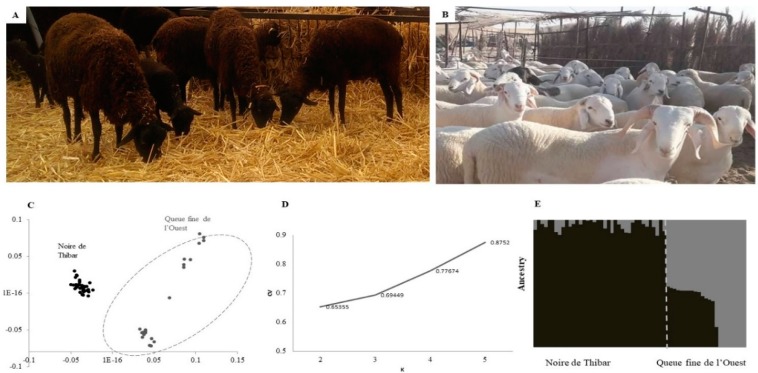
(**A**) Noire de Thibar animals; (**B**) A Queue fine de l’ouest flock; (**C**) Multidimensional scaling (MDS) plot of identity-by-state (IBS) matrix; (**D**) Plot of admixture cross-validation error with tested K from 2 to 5; (**E**) Admixture analysis using K = 2, representing the estimated level of admixture in each sheep population.

In the present study, 61 sheep samples belonging to 38 black Noire de Thibar (described in the introduction section; Figure 1A) and 23 Queue fine de l’ouest samples (Figure 1B) were genotyped for further analysis. In fact, the thin-tailed Queue fine de l’ouest is a white-coated, sheep meat breed found mostly in West–central Tunisia and originated from the Algerian Ouled Djellal breed. The samples were collected from animals without any specified diseases by veterinary clinics, but we assumed that white individuals were susceptible, while the black ones were assumed to be photosensitization-tolerant, as suggested by previous reports about the Tunisian Noire de Thibar sheep [6,9,10].

### 2.3. Genotyping-by-Sequencing, SNP Calling and Filtering

Genomic DNA was isolated from blood samples using the standard phenol/chloroform protocol [21]. The A260/280 ratios of DNA samples were determined with NanoDrop 2000 (Thermo Scientific, Weltham, MA, USA). The samples with an A260/280 ratio between 1.7 and 2.0 were genotyped using the genotyping-by-sequencing (GBS) protocol at the AgResearch Invermay Research Centre, Invermay, New Zealand. GBS library preparation and purification was performed, as described in Reference [22], using PstI/MspI restriction enzymes, following the method outlined by Elshire et al. [23], and sequencing was performed on an Illumina HiSeq2500 utilizing v4 chemistry (Illumina, San Diego, CA, USA) and 100 bp single end reads.

Raw fastq files were quality checked using FastQCv0.10.1 (http://www.bioinformatics.babraham.ac.uk/projects/fastqc/). SNPs were detected using default UNEAK (Universal Network-Enabled Analysis Kit) (Cornell University, New York, NY, USA) [24] pipeline parameters implemented in TASSEL 3.0 software. Briefly, good reads were defined as reads carrying a perfect barcode match with no Ns in the 64-bp following the barcode. Reads were subsequently trimmed to 64-bp (excluding barcodes) are collapsed into TAGs (identical 64-bp reads). Unique 64-bp, which were present five or more times across all samples, were retained and used to identify “TAG pairs” (pairs having a single base pair mismatch) with a default error tolerance rate (ETR) of 0.03, as described in Reference [24]. Reciprocal “TAG pairs” with only 1-bp mismatch were considered putative SNPs. The reliability and consistency of the genotyping data were evaluated using a quality control analysis pipeline: “Deconvolute and quality control” (https://github.com/AgResearch/DECONVQC). Tags were mapped onto sheep reference genome *Ovis_aries.* Oar_v3.1 using a bowtie2.2.3.0 sequence aligner using standard settings in order to generate whole genome SNP data. Quality filters were applied to discard low-quality markers, and thus, all SNPs were additionally filtered to remove those with minor allele frequencies (MAFs) lower than 0.05, SNP call rates lower than 90%, and deviation from the Hardy–Weinberg equilibrium with *p* < 0.001. Only SNPs located on autosomes were considered in further analyses. Moreover, individuals with >10% of missing genotypes were also removed.

### 2.4. Population Stratification

Pair-wise IBS allele sharing estimates among sheep samples were calculated using PLINK 1.7 [25] and were graphically represented by an MDS plot. The admixture level of each animal in two sheep populations was estimated using a model-based clustering algorithm implemented in the software ADMIXTURE v1.2.3 [26], assuming two parental populations (*K* = 2) after calculating the most appropriate K value using the admixture’s cross validation error in different K values ranging from two to five (Figure 1D).The unsupervised method applied a maximum likelihood-based clustering algorithm that placed individuals into two predefined clusters without prior population information.

### 2.5. Detection of Selection Signals

After the sheep population differentiation was estimated (see results), we considered two breeds as case/control groups to obtain the Wright’s F_ST_ estimate for each SNP, via Weir and Cockerham’s method [27] using the—fst option in PLINK1.9 [25,28]. In order to detect superior signals based on the allele frequency difference, markers were ranked according to raw F_ST_ values and plotted according to their genomic position based on the *Ovis_aries*Oar_v3.1 assembly genome [29] using R software (version 2.13.2).

The top 0.1% ranked SNPs (n = 40) were considered as putative markers. SNP-containing or the nearest annotated genes for each potential marker were considered as putative genomic regions under selection. Annotated genes were obtained from the NCBI sheep genome data viewer version 4.8.3 (https://www.ncbi.nlm.nih.gov/genome/gdv/browser/gene/?id=101104604). Gene functions were determined using NCBI (http://www.ncbi.nlm.nih.gov/gene/) and were supported by an extensive literature search. Genes located at less than 200kb away from the potential SNPs were considered candidate genes. Moreover, the linkage disequilibrium (LD) structure and haplotypes blocks were defined using HAPLOVIEW v4.2 [30].

## 3. Results

### 3.1. Population Differentiation

After DNA sequencing, SNP discovery yielded ~116,000 sequence tags with a mean depth of 3.33. Of these, 100,333 SNPs were then successfully mapped to the genome and 39,788 GBS markers, genotyped in 61 sheep samples, were retained after data pruning and were used for further analysis. The characteristics of filtered SNPs were mentioned in Table S1. When considering the average call rate for the filtered SNPs set, 97% of markers had assigned genotypes with an average minor allele frequency (MAF) of 0.23 and an average observed heterozygosity 0.25.Population structure was assessed using 38 black-coated Noire de Thibar (Figure 1A) and 23 white-coated Queue fine de l’ouest samples (Figure 1B).The results of the population structure, provided by MDS (Figure 1C), showed two clearly differentiated groups. In fact, Noire de Thibar animals were grouped in one tight cluster and were isolated from Queue fine de l’ouest samples. The ADMIXTURE method allowed for the assignment of individuals to groups based on their genetic similarities. Therefore, based on the results of the ADMIXTURE cross-validation analysis, the most likely value, K, was 2 (Figure 1D), suggesting that two breeds clustered consistently with MDS results, showing different ancestral genetic backgrounds, with the exception of some probably admixed Queue fine de l’ouest animals (Figure 1E).

### 3.2. Detection of F_ST_ Outlier Loci

**Figure 2:**
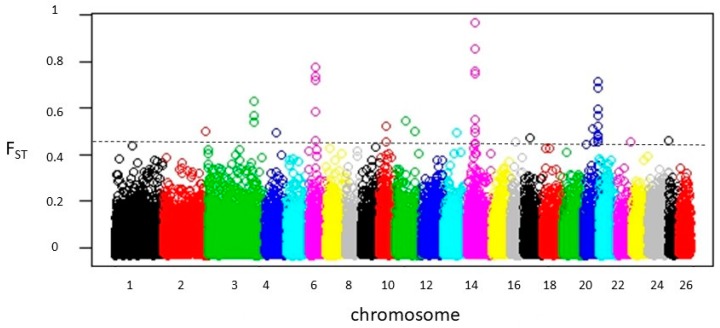
Genome-wide distribution of F*_ST_* values for pair-wise comparison of Noire de Thibar vs. Queue fine de l’ouest sheep breeds. GBS markers were ordered according to their genomic position through 26 autosomes. The black dashed line represents the threshold of significance (top 0.1% SNPs; F_ST_ = 0.45).

**Figure 3:**
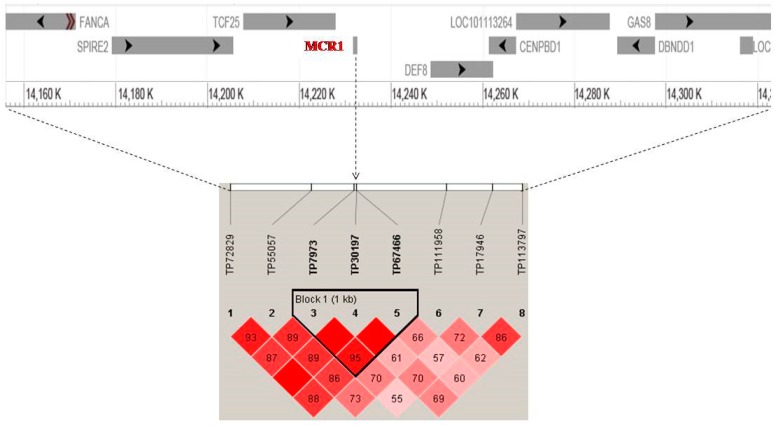
Linkage disequilibrium plot between eight significant SNPs on chromosome 14. The plot reveals a high Linkage disequilibrium (LD) block spanning 1Kb around the most significant SNP *chr14*:14231897 within and near the *MC1R* gene. Gene track images are from NCBI Genome Data Viewer (https://www.ncbi.nlm.nih.gov/genome/gdv/browser/?context=genome&acc=GCF_000298735.1).

The results revealed 40 potential SNPs distributed on 17 differentiated genomic regions with F_ST_ values ranging from 0.45 to 0.97 (Figure 2; Table S2; Table 1). Table S2 also lists the details of the potential SNP markers and genes found within putative genomic regions. The results identify genomic regions on chromosomes 3, 6, 14, and 20 (Table 1), where the related significance markers were positioned no further than 200 Kb apart (Table S2). Therefore, the length of the genomic regions under selection ranged from 400 Kb (within one significant SNP) to ~610 Kb in region 3 on chromosome (OAR) 20 (Table 1). The genome-wide distribution of F_ST_ values, represented in Figure 2, shows that the highest signal of selection was located in region 1, containing eight potential SNPs on chromosome 14 (Average F_ST_ = 0.67) where the most significant locus (*chr14*:14231897; F_ST_ = 0.97) was positioned at position 14.23 Mb, overlapping with the *MC1R* gene. In addition, the linkage disequilibrium (LD) plot (Figure 3) of eight significant SNP s most likely tracked the same quantitative trait loci (QTL), which is the *MC1R* gene in the high LD block with (*chr 14*: 14230826, 14231897, and 14232016) markers. The second significant signal was located on OAR 6, containing five consecutive significance markers with peak SNP (*chr6*:70008012; F_ST_ = 0.87) spanning 487.267 Kb and harboring five potential genes, among them, the *PDGFRA* and *KIT* genes. The third genomic region, located on OAR 20, represented the broadest genomic region with 10 consecutive SNPs spanning 610,843 Kb and harboring eight genes at approximately 60 Kb upstream *EXOC2* and *IRF4* genes.

## 4. Discussion

### High Signals of Selection in Pigmentation Candidate Genes

Estimates of the population structure were in accordance with F_ST_ at individual loci, which showed a high level of heterogeneity across the genome. Indeed, the genetic differentiation between the two breeds was shaped by the introgression of European Merino-type gene flow into the Noire de Thibar breed, coupled with intensive selective pressure on coat color [7,31]. Selection of favorable variants is expected to result in a higher level of differentiation for neighboring SNPs. In several instances, outlier SNPs tended to cluster in similar regions (e.g., OAR 3, OAR6, OAR 14, and OAR 20). The observed pattern of region differentiation was probably due to the high linkage-disequilibrium of dense GBS markers. Chang et al. [32] reported that using high density SNP markers was sufficient to detect the LD between markers and causative mutations. Thus, our results are in agreement with previous findings reporting that when a beneficial mutation emerges and subsequently spreads in a population, this process, which is a selective sweep [33], will generate higher population differentiation, higher frequencies of segregating sites, and a characteristic linkage disequilibrium (LD) pattern [34]. Interestingly, as expected, five genomic regions among the seventeen identified in this study harbored key genes related to the pigmentation trait in sheep. This is not unexpected, given that the photosensitization can be the most severe in any portion of the animal exposed to sunlight that lacks a protective fleece, hair, or pigmentation [35,36,37]. The most significant genomic region under selection wasregion1, located on OAR 14, containing the Melanocortin 1 receptor *MC1R* gene, a key candidate gene for coat color pigmentation. Indeed, the *MC1R* gene played a central role in the regulation of eumelanin (black-brown) and phaeomelanin (red-yellow) synthesis within the mammalian melanocyte and is often found under selection in several sheep breeds [38,39]. Moreover, previous sequence analysis studies evidenced those mutations, within the *MC1R* gene, which are responsible for the dominant black-color in sheep [40,41] and pigs [42]. In addition, it could play an important role in the prevention of light-sensitive diseases. To support this, several recessive genetic loss-of-function variants in the *MC1R* gene increase the risk of developing cutaneous melanoma [43] and have been found to be associated to phenotypic features related to sun sensitivity in European populations [44].The high signal in region 2, located on OAR 6, harbored the *KIT* and platelet derived growth factor receptor alpha *PDGFRA* genes, which are implicated and interact in the functional pathway of coat color in different mammals. For example, the gene complexes *KDR*, *KIT*, and *PDGFRA* were found to be associated with the reddening coat color pattern in Angus cattle [45]. In addition, *KIT* and *PDGFRA* were assumed to play important roles in determining white-coat color in Iranian goats [46]. Genomic region 3 on OAR 20 spans at position (50,143,014–50,753,857), located close to *IRF4* gene (50,969,622–50,985,660), and is associated with skin pigmentation, hair color, or skin sensitivity to the sun in humans [47,48,49] and goats [50]. Indeed, the *IRF4* gene has been implicated in melanocytic biology; the *IRF4* protein was proposed as a diagnostic marker for various melanoma subtypes. Moreover, the transcription factors *SOX-10* and *PICK1* genes, located on OAR 3, were just downstream of region 4. *SOX-10* has been identified as a selection signature responsible for coat pigmentation in sheep [51,52] and *PICK1* has a well-established role in melanocyte biology and is essential for melanocyte migration and survival in the plumage color in chickens [53]. Similarly, the endothelin 3 or *EDN3* gene (OAR1: 56,388,076-56,412,489), located downstream of the *ZNF831* gene, was also detected in studies as the footprint of the selection pigmentation trait in Awassi [51] and worldwide sheep breeds [52].

## 5. Conclusions

These findings demonstrate the efficiency of genotyping-by-sequencing (GBS) data, even with a relatively low number of animals, to identify signals of selection for pigmentation candidate genes in two local Tunisian sheep breeds. The variants underlying these signals could play a potential role in adaptation to photosensitive symptoms caused by toxic weeds in white sheep. However, it will be necessary to carry out association and functional studies to demonstrate the implication of these genes’ in tolerance to sheep-photosensitization, and therefore, their efficiency as future marker-assisted selection.

## Figures and Tables

**Table 1:** Genomic regions and associated candidate genes related to their pigmentation trait.

Region	OAR	Significant SNP		Genomic Region			
		F_ST_^1^	Position (pb) ^2^	Markers ^3^	Length (Kb)	Genes ^4^	Candidate Genes
1	14	0.97	14231897	8	576	24	*MC1R*
2	6	0.78	70008012	5	487	5	*PDGFRA, KIT*
3	20	0.72	50553857	10	611	8	*IRF4, PICK1*
4	3	0.63	213795317	3	416	17	*SOX10, PICK1*
5	20	0.54	38835509	1	400	8	*-*
6	11	0.52	32377178	1	400	7	*-*
7	10	0.51	35837558	1	400	14	*-*
8	13	0.51	56477753	1	400	6	*EDN3*
9	4	0.50	97859387	1	400	8	-
10	11	0.49	53654935	1	400	8	-
11	2	0.48	247265800	1	400	4	-
12	20	0.46	44022745	1	400	5	-
13	17	0.46	44834726	1	400	7	-
14	10	0.46	36042096	1	400	8	-
15	22	0.46	50353516	1	400	10	-
16	25	0.45	1327751	1	400	1	-
17	16	0.45	28645498	1	400	5	-

^1^ the peak SNPs within each region were ranked according to their F_ST_ value; ^2^ markers are positioned using sheep genome assembly version 3.1; ^3^ Number of consecutive markers or those with physical distance < 200Kb; ^4^ number of genes within the enriched genomic region under selection.

## Supplementary Materials

The following are available online at https://www.mdpi.com/2076-2615/10/1/5/s1, Table S1: Characteristics of final filtered SNPs (39,788 GBS markers), Table S2: Details about putative SNPs under selection and related differentiated genomic region using F_ST_ estimates using pairwise comparison between Noire de Thibar and Queue fine de l’ouest breeds.

## References

[1] Kijas J.W., Lenstra J.A., Hayes B., Boitard S., Neto L.R.P., San Cristobal M., Servin B., McCulloch R., Whan V., Gietzen K. (2012). Genome-wide analysis of the world’s sheep breeds reveals high levels of historic mixture and strong recent selection. PLoS Biol..

[2] Rowe L.D. (1989). Photosensitization problems in livestock. Vet. Clin. North Am. Food Anim. Pract..

[3] Schempp C., Lüdtke R., Winghofer B., Simon J. (2000). Effect of topical application of Hypericum perforatum extract (St. John’s wort) on skin sensitivity to solar simulated radiation. Photodermatol. Photoimmunol. Photomed..

[4] Quinn J.C., Kessell A., Weston L.A. (2014). Secondary plant products causing photosensitization in grazing herbivores: Their structure, activity and regulation. Int. J. Mol. Sci..

[5] Schempp C., Müller K., Winghofer B., Schöpf E., Simon J. (2002). St. John’s wort (Hypericum perforatum L.). A plant with relevance for dermatology. Der Hautarzt Z. Dermatol. Venerol. Verwandte Geb..

[6] Kallal A. (1968). Lemoutonnoirde Thibar. Ph.D. Thesis.

[7] Chalh A., El Gazzah M., Djemali M., Chalbi N. (2007). Genetic and phenotypic characterization of the Tunisian Noire De Thibar lambs on their growth traits. J. Biol. Sci..

[8] Rekik M., Aloulou R., Hamouda B.M. (2005). Small ruminant breeds of Tunisia. Charact. Small Rumin. Breeds West Asia North Afr..

[9] Bedhiaf-Romdhani S., Djemali M., Zaklouta M., Iniguez L. (2008). Monitoring crossbreeding trends in native Tunisian sheep breeds. Small Rumin. Res..

[10] Djemali M. Genetic improvement objectives of sheep and goats in Tunisia. Lessons learned. Proceedings of the Options Méditerranéennes, Série ASéminaires Méditerranéens.

[11] Koseniuk A., Ropka-Molik K., Rubiś D., Smołucha G. (2018). Genetic background of coat colour in sheep. Arch. Anim. Breed..

[12] Solano F. (2014). Melanins: Skin pigments and much more—types, structural models, biological functions, and formation routes. New J. Sci..

[13] Hernando B., Sanz-Page E., Pitarch G., Mahiques L., Valcuende-Cavero F., Martinez-Cadenas C. (2018). Genetic variants associated with skin photosensitivity in a southern European population from Spain. Photodermatol. Photoimmunol. Photomed..

[14] Khalesi M., Whiteman D.C., Tran B., Kimlin M.G., Olsen C.M., Neale R.E. (2013). A meta-analysis of pigmentary characteristics, sun sensitivity, freckling and melanocytic nevi and risk of basal cell carcinoma of the skin. Cancer Epidemiol..

[15] Gurgul A., Miksza-Cybulska A., Szmatoła T., Jasielczuk I., Piestrzyńska-Kajtoch A., Fornal A., Semik-Gurgul E., Bugno-Poniewierska M. (2019). Genotyping-by-sequencing performance in selected livestock species. Genomics.

[16] Wang Z., Ma H., Xu L., Zhu B., Liu Y., Bordbar F., Chen Y., Zhang L., Gao X., Gao H. (2019). Genome-wide scan identifies selection signatures in chinese wagyu cattle using a high-density SNP array. Animals.

[17] Wang K., Wu P., Yang Q., Chen D., Zhou J., Jiang A., Ma J., Tang Q., Xiao W., Jiang Y. (2018). Detection of selection signatures in Chinese Landrace and Yorkshire pigs based on genotyping-by-sequencing data. Front. Genet..

[18] Wu P., Yang Q., Wang K., Zhou J., Ma J., Tang Q., Jin L., Xiao W., Jiang A., Jiang Y. (2018). Single step genome-wide association studies based on genotyping by sequence data reveals novel loci for the litter traits of domestic pigs. Genomics.

[19] Pértille F., Zanella R., Felício A., Ledur M., PEIXOTO J.d.O., Coutinho L.L. (2015). Identification of polymorphisms associated with production traits on chicken (*Gallus gallus*) chromosome 4. Embrapa Suínos E Aves-Artig. Em Periódico Indexado (Alice).

[20] Bahbahani H., Musa H.H., Wragg D., Shuiep E.S., Almathen F., Hanotte O. (2019). Genome diversity and signatures of selection for production and performance traits in dromedary camels. Front. Genet..

[21] Sambrook J., Fritsch E.F., Maniatis T. (1989). Molecular Cloning: A laboratory Manual.

[22] Dodds K.G., McEwan J.C., Brauning R., Anderson R.M., van Stijn T.C., Kristjánsson T., Clarke S.M. (2015). Construction of relatedness matrices using genotyping-by-sequencing data. BMC Genom..

[23] Elshire R.J., Glaubitz J.C., Sun Q., Poland J.A., Kawamoto K., Buckler E.S., Mitchell S.E. (2011). A robust, simple genotyping-by-sequencing (GBS) approach for high diversity species. PLoS ONE.

[24] Lu F., Lipka A.E., Glaubitz J., Elshire R., Cherney J.H., Casler M.D., Buckler E.S., Costich D.E. (2013). Switchgrass genomic diversity, ploidy, and evolution: Novel insights from a network-based SNP discovery protocol. PLoS Genet..

[25] Purcell S., Neale B., Todd-Brown K., Thomas L., Ferreira M.A., Bender D., Maller J., Sklar P., De Bakker P.I., Daly M.J. (2007). PLINK: A tool set for whole-genome association and population-based linkage analyses. Am. J. Hum. Genet..

[26] Alexander D.H., Lange K. (2011). Enhancements to the ADMIXTURE algorithm for individual ancestry estimation. BMC Bioinform..

[27] Weir B.S., Cockerham C.C. (1984). Estimating F-statistics for the analysis of population structure. Evolution.

[28] Chang C.C., Chow C.C., Tellier L.C., Vattikuti S., Purcell S.M., Lee J.J. (2015). Second-generation PLINK: Rising to the challenge of larger and richer datasets. Gigascience.

[29] Jiang Y., Xie M., Chen W., Talbot R., Maddox J.F., Faraut T., Wu C., Muzny D.M., Li Y., Zhang W. (2014). The sheep genome illuminates biology of the rumen and lipid metabolism. Science.

[30] Barrett J.C., Fry B., Maller J., Daly M.J. (2004). Haploview: Analysis and visualization of LD and haplotype maps. Bioinformatics.

[31] Sassi-Zaidy Y.B., Maretto F., Charfi-Cheikrouha F., Cassandro M. (2014). Genetic diversity, structure, and breed relationships in Tunisian sheep. Small Rumin. Res..

[32] Chang L.-Y., Toghiani S., Ling A., Aggrey S.E., Rekaya R. (2018). High density marker panels, SNPs prioritizing and accuracy of genomic selection. BMC Genet..

[33] Smith J.M., Haigh J. (1974). The hitch-hiking effect of a favourable gene. Genet. Res..

[34] Grossman S.R., Shylakhter I., Karlsson E.K., Byrne E.H., Morales S., Frieden G., Hostetter E., Angelino E., Garber M., Zuk O. (2010). A composite of multiple signals distinguishes causal variants in regions of positive selection. Science.

[35] Gupta R.C. (2012). Veterinary Toxicology: Basic and Clinical Principles.

[36] Fu P.P., Xia Q., Zhao Y., Wang S., Yu H., Chiang H.-M. (2013). Phototoxicity of herbal plants and herbal products. J. Environ. Sci. HealthPart C.

[37] Chen Y., Quinn J.C., Weston L.A., Loukopoulos P. (2019). The aetiology, prevalence and morbidity of outbreaks of photosensitisation in livestock: A review. PLoS ONE.

[38] Li J., Yang H., Li J., Li H., Ning T., Pan X., Shi P., Zhang Y. (2010). Artificial selection of the melanocortin receptor 1 gene in Chinese domestic pigs during domestication. Heredity.

[39] Gouveia J.J.d.S., Silva M.V.G.B.d., Paiva S.R., Oliveira S.M.P.d. (2014). Identification of selection signatures in livestock species. Genet. Mol. Biol..

[40] Kijas J., Serrano M., McCulloch R., Li Y., Salces Ortiz J., Calvo J., Pérez-Guzmán M., Consortium I.S.G. (2013). Genomewide association for a dominant pigmentation gene in sheep. J. Anim. Breed. Genet..

[41] Fontanesi L., Dall’Olio S., Beretti F., Portolano B., Russo V. (2011). Coat colours in the Massese sheep breed are associated with mutations in the agouti signalling protein (*ASIP*) and melanocortin 1 receptor (*MC1R*) genes. Animal.

[42] Kijas J., Moller M., Plastow G., Andersson L. (2001). A frameshift mutation in *MC1R* and a high frequency of somatic reversions cause black spotting in pigs. Genetics.

[43] Kennedy C., ter Huurne J., Berkhout M., Gruis N., Bastiaens M., Bergman W., Willemze R., Bavinck J.N.B. (2001). Melanocortin 1 receptor (*MC1R*) gene variants are associated with an increased risk for cutaneous melanoma which is largely independent of skin type and hair color. J. Investig. Dermatol..

[44] Latreille J., Ezzedine K., Elfakir A., Ambroisine L., Gardinier S., Galan P., Hercberg S., Gruber F., Rees J., Tschachler E. (2009). *MC1R* gene polymorphism affects skin color and phenotypic features related to sun sensitivity in a population of French adult women. Photochem. Photobiol..

[45] Hanna L.L.H., Sanders J.O., Riley D.G., Abbey C.A., Gill C.A. (2014). Identification of a major locus interacting with *MC1R* and modifying black coat color in an F_2_ Nellore-Angus population. Genet. Sel. Evol..

[46] Nazari-Ghadikolaei A., Mehrabani-Yeganeh H., Miarei-Aashtiani S.R., Staiger E.A., Rashidi A., Huson H.J. (2018). Genome-wide association studies identify candidate genes for coat color and mohair traits in the iranian markhoz goat. Front. Genet..

[47] Sulem P., Gudbjartsson D.F., Stacey S.N., Helgason A., Rafnar T., Magnusson K.P., Manolescu A., Karason A., Palsson A., Thorleifsson G. (2007). Genetic determinants of hair, eye and skin pigmentation in Europeans. Nat. Genet..

[48] Han J., Kraft P., Nan H., Guo Q., Chen C., Qureshi A., Hankinson S.E., Hu F.B., Duffy D.L., Zhao Z.Z. (2008). A genome-wide association study identifies novel alleles associated with hair color and skin pigmentation. PLoS Genet..

[49] Praetorius C., Grill C., Stacey S.N., Metcalf A.M., Gorkin D.U., Robinson K.C., Van Otterloo E., Kim R.S., Bergsteinsdottir K., Ogmundsdottir M.H. (2013). A polymorphism in IRF4 affects human pigmentation through a tyrosinase-dependent MITF/TFAP2A pathway. Cell.

[50] Guo J., Tao H., Li P., Li L., Zhong T., Wang L., Ma J., Chen X., Song T., Zhang H. (2018). Whole-genome sequencing reveals selection signatures associated with important traits in six goat breeds. Sci. Rep..

[51] Seroussi E., Rosov A., Shirak A., Lam A., Gootwine E. (2017). Unveiling genomic regions that underlie differences between Afec-Assaf sheep and its parental Awassi breed. Genet. Sel. Evol..

[52] Fariello M.-I., Servin B., Tosser-Klopp G., Rupp R., Moreno C., San Cristobal M., Boitard S., Consortium I.S.G. (2014). Selection signatures in worldwide sheep populations. PLoS ONE.

[53] Gunnarsson U., Kerje S., Bed’hom B., Sahlqvist A.S., Ekwall O., Tixier-Boichard M., Kämpe O., Andersson L. (2011). The Dark brown plumage color in chickens is caused by an 8.3-kb deletion upstream of *SOX10*. Pigment Cell Melanoma Res..

